# Association of Urinary Dickkopf-3 with Residual Renal Function Decline in Patients Undergoing Peritoneal Dialysis

**DOI:** 10.3390/medicina57060631

**Published:** 2021-06-18

**Authors:** Kenta Torigoe, Kumiko Muta, Kiyokazu Tsuji, Ayuko Yamashita, Miki Torigoe, Shinichi Abe, Yuki Ota, Hiroshi Mukae, Tomoya Nishino

**Affiliations:** 1 Department of Nephrology, Nagasaki University Hospital, 1-7-1 Sakamoto, Nagasaki 852-8501, Japan; ktorigoe@nagasaki-u.ac.jp (K.T.); tsuji-kiyo@nagasaki-u.ac.jp (K.T.); ayamashita@nagasaki-u.ac.jp (A.Y.); sawamiki.114@gmail.com (M.T.); s-abe@nagasaki-u.ac.jp (S.A.); yukiota@hospital.sasebo.nagasaki.jp (Y.O.); tnishino@nagasaki-u.ac.jp (T.N.); 2 Department of Respiratory Medicine, Nagasaki University Graduate School of Biomedical Sciences, 1-7-1 Sakamoto, Nagasaki 852-8501, Japan; hmukae@nagasaki-u.ac.jp

**Keywords:** biomarker, Dickkopf-3, peritoneal dialysis, residual renal function

## Abstract

*Background and Objectives*: Urinary levels of dickkopf-3 (DKK-3) are associated with poor renal survival in patients with non-dialytic chronic kidney disease. However, it remains unknown whether urinary DKK-3 levels can predict residual renal function (RRF) decline in patients undergoing peritoneal dialysis (PD). Therefore, we investigated the correlation between urinary levels of DKK-3 and the subsequent rate of RRF decline in PD patients. *Materials and Methods*: This study included 36 PD patients who underwent multiple peritoneal equivalent tests during 2011–2021. The relationship between baseline clinical characteristics and the subsequent annual rate of Kt/V decline was investigated. *Results*: The annual rate of renal Kt/V decline was 0.29 (range: 0.05–0.48), which correlated with renal Kt/V (*r* = 0.55, *p* = 0.0005) and 24 h urinary DKK-3 excretion (*r* = 0.61, *p* < 0.0001). Similarly, 24 h urinary DKK-3 excretion (β = 0.44, *p* = 0.0015) and renal Kt/V (β = 0.38, *p* = 0.0059) were independently associated with the annual rate of renal Kt/V decline in multivariate analyses. *Conclusions*: Urinary DKK-3 assessment may help identify PD patients at a high risk of RRF decline.

## 1. Introduction

Chronic kidney disease (CKD) is a common public health problem with an estimated global prevalence of 13.4% [1]. CKD progression leads to end-stage renal disease (ESRD), which requires treatment with renal replacement therapy (RRT).

Peritoneal dialysis (PD) and hemodialysis (HD) are the major types of RRT for patients with ESRD. In both cases, residual renal function (RRF) is a key factor associated with patient survival [2,3,4]. Compared with HD, PD is associated with better preservation of RRF [5]; however, RRF loss in PD patients has been associated with withdrawal from PD [6]. In addition, RRF loss is associated with poor quality of life and worsening fluid status in PD patients [7,8]. Therefore, preserving RRF is a critical issue for PD patients. Previous studies have shown that some urinary biomarkers, including proteinuria, can predict RRF decline in PD patients [9,10,11,12]. However, there is no established marker for predicting RRF decline in PD patients. The identification of patients at high risk of RRF decline could enable early intervention of RRF preservation; thus, new biomarkers for predicting an increased RRF decline in PD patients are desired.

Dickkopf-3 (DKK-3) is a renal tubular secreted glycoprotein induced by various tubular stresses, which stimulates tubulointerstitial fibrosis by affecting the Wnt/β-catenin signaling pathway [13]. In the clinical setting, higher urinary DKK-3 levels predict poor renal survival in various cases of non-dialytic CKD [14]. Furthermore, urinary DKK-3 is associated with acute kidney injury and subsequent chronic kidney dysfunction in patients undergoing cardiac surgery [15]. Urinary DKK-3 levels are a promising marker for predicting RRF decline and assessing the effects of interventions in various kidney diseases [16,17]. However, there are no data on whether urinary levels of DKK-3 are useful for predicting RRF decline in PD patients. Therefore, in this study, we investigated the correlation between urinary levels of DKK-3 and the subsequent rate of RRF decline in PD patients.

## 2. Materials and Methods

This was a retrospective, longitudinal, observational cohort study that included 36 PD patients. All PD patients underwent a peritoneal equilibration test (PET) at least twice between October 2011 and March 2021. Patients with anuria (daily urine volume < 200 mL) or those who underwent a combination therapy with concurrent PD and HD at the time of the first PET were excluded. This study was conducted in accordance with the Declaration of Helsinki and the ethical guidelines for medical and health research involving human subjects. The ethics review board of Nagasaki University Hospital approved the study protocol (approval number: 09022759-5).

### 2.1. Date and Sample Collection

The patient’s baseline data and 24 h urine samples were simultaneously collected at the time of the first PET. The results of PET were calculated using the PD ADEQUEST 2.0 software (Baxter International Inc., Deerfield, IL, USA). In this study, weekly renal Kt/V urea, which reflects the rate of residual renal urea clearance, was used as a marker of RRF. The annual rate of renal Kt/V decline was calculated according to the following formula:

(renal Kt/V at first PET–renal Kt/V at the next PET)/number of follow-up years

### 2.2. Measurement of Urinary DKK-3

We analyzed urinary DKK-3 levels using an enzyme-linked immunosorbent assay (ELISA) kit from 24 h urine samples collected at the time of the first PET. The Human DKK-3 ELISA Kit (RAB0145; Sigma-Aldrich, Saint Louis, MO, USA) was used according to the manufacturer’s instructions. Absorbance was measured at 450 nm using a microplate reader (MULTISKAN FC; Thermo Scientific, Kanagawa, Japan). All samples were measured in duplicate.

### 2.3. Statistical Analysis

Categorical variables are expressed as percentages. Normally distributed continuous variables are expressed as mean ± standard deviation, and non-normally distributed variables are expressed as median and interquartile range. Pearson’s correlation test and multiple linear regression analysis for renal Kt/V decline were performed using the variables that were reported as predictors of RRF decline [18], including mean arterial pressure, serum albumin, total-cholesterol, renal Kt/V, total Kt/V, 4h-D/Pcr, urinary protein, urinary DKK-3, diabetes mellitus, administration of ACE-I/ARB and diuretics, and history of peritonitis. Regarding multivariate linear regression analysis, considering the study sample size, the Akaike information criterion (AIC) was used to exclude variables that did not improve the model. The model with the lowest AIC score was used in multiple linear regression analysis. Statistical analyses were performed using JMP version 14 software (SAS Institute Inc., Cary, NC, USA). Statistical significance was set at *p* < 0.05.

## 3. Results

The characteristics of PD patients at the time of the first PET are presented in Table 1. The mean age of the patients was 61.1 ± 12.8 years, and 21 (55.6%) patients were men. The median duration of PD at baseline was 13 (range: 11–24) months. The main cause of ESRD was nephrosclerosis (33.3%). The median urinary volume was 1500 (range: 1140–1700) mL/day, and the mean fluid removal rate was 248 ± 368.6 mL/day. The median total Kt/V was 1.95 (range: 1.63–2.48), which suggested sufficient small molecule clearance. The median 24 h urinary DKK-3 excretion rate was 13.4 (range, 4.1–28.2) μg/day. The median renal Kt/V was 0.84 (range: 0.64–1.40), suggesting that the RRF was relatively preserved in patients undergoing PD. The annual rate of renal Kt/V decline in PD patients was 0.29/year (range: 0.05–0.48), measured at the median follow-up period of 11.5 (range: 11–12) months.

### Factors Associated with RRF Decline in PD Patients

Next, we investigated the factors that correlated with the annual rate of renal Kt/V decline in PD patients. As shown in Table 2, simple regression analysis revealed that renal Kt/V (*r* = 0.55, *p* = 0.0005), and 24 h urinary DKK-3 excretion (*r* = 0.61, *p* < 0.0001) were positively correlated with the annual rate of renal Kt/V decline. There was no correlation between the 24 h urinary protein excretion and the annual rate of renal Kt/V decline.

**Figure 1:**
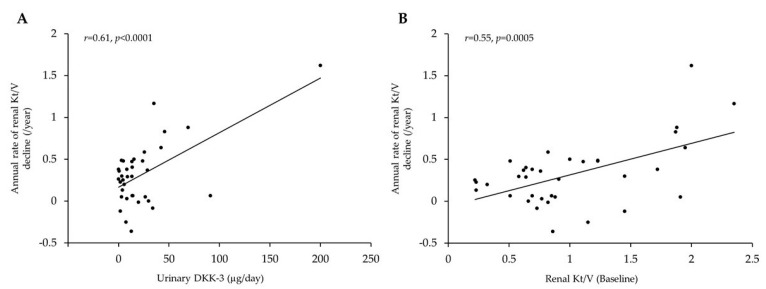
Relationship between residual renal function decline and patient characteristics. (**A**) Correlation between urinary DKK-3 levels and subsequence annual rate of renal Kt/V decline. (**B**) Correlation between baseline renal Kt/V and the subsequent annual rate of renal Kt/V decline.

Furthermore, to identify the independent factors that correlate with the annual rate of renal Kt/V decline, we performed multiple regression analyses using the minimum AIC score. Urinary protein, renal Kt/V, and 24 h urinary DKK-3 excretion were selected as independent variables for multiple regression analysis. Both renal Kt/V (β = 0.38, *p* = 0.0059) and daily urinary DKK-3 excretion rate (β = 0.44, *p* = 0.0015) were independently associated with a higher annual rate of renal Kt/V decline in PD patients (adjusted R^2^ = 0.55, *p* < 0.0001) (Table 2, Figure 1). Finally, the urinary DKK-3/creatinine ratio also correlated with the annual rate of renal Kt/V decline in simple linear regression analysis (*r* = 0.40, *p* = 0.0153).

## 4. Discussion

In this study, we investigated the correlation between urinary DKK-3 level and RRF decline in PD patients and demonstrated that higher 24-h urinary DKK-3 excretion was independently correlated with the annual rate of renal Kt/V decline.

Urinary DKK-3 is a novel biomarker of acute kidney injury and CKD [14,15,19]. In particular, in non-dialytic CKD patients, urinary DKK-3 levels have been associated with CKD progression regardless of disease etiology [14]. Urinary DKK-3 is excreted by the tubular cells under stress and activates the Wnt/β-catenin signaling [16,17]. The Wnt/β-catenin signaling pathway is involved in organ development, tissue homeostasis, and human disease pathogenesis [20]. In kidney disease, the Wnt/β-catenin signaling pathway acts as an inducer of tubulointerstitial fibrosis by upregulating the expression of profibrotic factors such as fibronectin, fibroblast-specific protein-1, snail-1, matrix metalloproteinase-7, plasminogen activator inhibitor-1, and the components of the renin-angiotensin system [21]. Tubulointerstitial fibrosis results from several factors and is strongly associated with renal prognosis [22,23,24]. In fact, although this study included various causes of ESRD, a high urinary DKK-3 level was positively correlated with RRF decline. This evidence suggests that, in PD patients with high rates of urinary DKK-3 excretion, DKK-3 may activate profibrotic gene expression, accelerate the progression of tubulointerstitial fibrosis, and decrease RRF, independently of the underlying cause of ESRD. Screening for high levels of urinary DKK-3 may help identify PD patients at a higher risk of RRF decline.

Furthermore, urinary DKK-3 may be an inducer of RRF decline. Basic research has shown that abolishing tubular DKK-3 attenuates tubulointerstitial fibrosis; this evidence suggests that decreasing urinary levels of DKK-3 may reduce the rate of RRF decline. However, at present, it remains unclear what kind of intervention may reduce urinary DKK-3 levels in a clinical setting. In our study, we used 24 h urinary DKK-3 excretion as a marker; however, collecting 24 h urine samples may not be practical in the clinical context. The urinary DKK-3/creatinine ratio also correlates with the annual rate of renal Kt/V decline and may be easier to assess in this patient group.

Previous studies have reported that various factors are associated with decreased residual renal function in patients undergoing PD [18], and this study considered these factors for analysis. Among these factors, the baseline renal Kt/V value was independently correlated with the rate of RRF decline in this study. Previous studies have reported that higher baseline RRF values were associated with a greater risk of subsequent RRF decline in PD patients [11,12]. However, this finding can be explained by the lead time bias [12]. PD patients with higher baseline RRF values have a lower risk of developing anuria than their counterparts [25]. Therefore, it is unnecessary to consider that patients with higher baseline RRF values are at a higher risk of RRF decline.

Other biomarkers have been associated with RRF decline in PD patients, such as urinary protein levels [9,10,11,12]. However, the present study did not show any association between urinary protein levels and the rate of RRF decline. Previously, 1 g/day of proteinuria has been associated with a 13.2% increase in the risk of progression to anuria in patients with PD [12]. Moreover, in the present study, PD patients presented with low levels of urinary protein (0.50 g/day, range: 0.27–0.76 g/day), potentially affecting the present results.

In our analysis, we selected independent values based on a previous study [18]. However, besides the aforementioned factors, other factors have been reported as predictors of RRF decline. For instance, serum uric acid was reported as a predictor of RRF decline in PD patients [26]. Another report showed that oral sodium bicarbonate decreases RRF decline [27]. Furthermore, glucose exposure, fluid overload, older age, inadequate hypertension, and high protein intake were similarly reported as factors of RRF decline in PD patients [11,12,28]. In this study, urinary DKK-3 was independently associated with renal Kt/V. However, it is possible that factors that could not be assessed in this study affect the associations between urinary DKK-3 and RRF decline in PD patients, and further study is needed to clarify this.

The present findings suggest that urinary DKK-3 levels may be a useful biomarker in PD patients. However, this study has several limitations. First, this was a retrospective observational study with small sample size. Thus, the impact of residual confounding factors, as mentioned above, could not be evaluated, and the power of analyses may be insufficient. Second, residual renal Kt/V was used as a marker of RRF in this study. Renal Kt/V is a renal urea clearance marker, and urea is a representative small-molecule uremic toxin. Thus, renal Kt/V could not reflect middle-to-large-sized molecule clearance, and the present findings may not reflect the true RRF. Third, the annual rate of renal Kt/V decline was estimated over a relatively short period (11.5 months, range, 11.0–12.0 months). Thus, our results may not reflect long-term RRF decline risk. Fourth, the present study did not account for other tubular injury markers, precluding any discussions about whether urinary DKK-3 is superior to other markers at predicting RRF decline. Further studies are therefore required to address these limitations.

## 5. Conclusions

In conclusion, we demonstrated that a higher 24 h urinary excretion of DKK-3 is positively correlated with the RRF decline rate in PD patients. Urinary DKK-3 assessment may help identify PD patients at a high risk of RRF decline.

## Figures and Tables

**Table 1:** Characteristics of peritoneal dialysis patients.

Characteristic	Value	Characteristic	Value
Age (years)	61.1 ± 12.8	Alb (g/dL)	3.3 ± 0.3
Duration of PD (months)	13 (11–24)	Corrected Ca (mg/dL)	9.2 ± 0.6
Male: Female	21:15	P (mg/dL)	5.2 (4.4–5.6)
BMI (kg/m^2^)	22.6 (20.3–25.3)	Intact-PTH (pg/mL)	167.8 (90.1–255.3)
MAP (mmHg)	92.9 ± 16.2	Total-cholesterol (mg/dL)	189.7 ± 34.2
History of peritonitis (%)	13.9%	HbA1c (%)	5.4 (5.1–5.7)
Primary disease of ESRD		CRP (mg/dL)	0.06 (0.03–0.21)
Chronic glomerulonephritis (%)	25.0	Urinary volume (mL/day)	1500 (1140–1700)
Diabetic kidney disease (%)	13.9	Effluent volume (mL/day)	248.0 ± 368.6
Nephrosclerosis (%)	33.3	Residual GFR (mL/min)	4.17 (2.60–6.51)
Other (%)	27.8	Peritoneal Kt/V	1.03 ± 0.37
Comorbidity		Renal Kt/V	0.84 (0.64–1.40)
Diabetes mellitus (%)	25.0	Total Kt/V	1.95 (1.63–2.48)
Charlson Comorbidity Index	3 (2–3)	4 h-D/P cr	0.61 (0.56–0.68)
ACE-I/ARB (%)	66.7	nPCR	0.88 ± 0.18
CCB (%)	63.9	Urinary protein (g/day)	0.50 (0.27–0.76)
Diuretic (%)	83.3	Urinary DKK-3 (μg/day)	13.4 (4.1–28.2)
Statin (%)	27.8	Renal Kt/V decline rate (/year)	0.29 (0.05–0.48)
CAPD: APD	24: 12	Duration of follow-up (month)	11.5 (11.0–12.0)
Hb (g/dL)	11.6 ± 1.4		

ACE-I: angiotensin-converting enzyme inhibitor; Alb: albumin; APD: automated peritoneal dialysis; ARB: angiotensin II receptor blocker; BMI: body mass index; BP: blood pressure; Ca: calcium; CAPD: continuous ambulatory peritoneal dialysis; CCB: calcium channel blocker; CRP: C-reactive protein; DKK-3: Dickkopf-3; GFR: glomerular filtration rate; Hb: hemoglobin; MAP: mean arterial pressure; nPCR: normalized protein catabolic rate; P: phosphorus; PD: peritoneal dialysis; PTH: parathyroid hormone; 4 h-D/P cr: 4 h dialysate/plasma creatinine.

**Table 2:** Pearson’s correlation analyses and multiple regression analyses for the rate of residual renal function decline in peritoneal dialysis patients.

	Pearson’s Correlation Analyses		Multiple Regression Analyses			
	*r*	*p*-Value	B	95% CI	β	*p*-Value
MAP (mmHg)	0.05	0.77		-		
Alb (g/dL)	0.23	0.18		-		
Total-cholesterol (mg/dL)	−0.05	0.78		-		
Renal Kt/V	0.55	0.0005	0.259	0.080–0.437	0.38	0.0059
Total Kt/V	0.32	0.06		-		
4 h-D/P cr	−0.19	0.27		-		
Urinary protein (g/day)	0.28	0.1	0.164	−0.011–0.338	0.23	0.0652
Urinary DKK-3 (μg/day)	0.61	<0.0001	0.005	0.002–0.008	0.44	0.0015
Diabetes mellitus				-		
ACE-I/ARB				-		
Diuretics				-		
History of peritonitis				-		

ACE-I: angiotensin-converting enzyme inhibitor; Alb: albumin; ARB: angiotensin II receptor blocker; B: unstandardized regression coefficient; β: standardized regression coefficient; CI: confidence interval; DKK-3: Dickkopf-3; MAP: mean arterial pressure; *r*: Pearson’s correlation coefficient; SE: standard error; 4 h-D/P cr: 4-h dialysate/plasma creatinine. Covariates not retained in the multivariate model with minimum Akaike information criterion are expressed as “-”.

## Data Availability

The data presented in this study are available on request from the corresponding author.

## References

[1] Coresh J. (2017). Update on the burden of CKD. J. Am. Soc. Nephrol..

[2] Wang A.Y., Lai K.N. (2006). The importance of residual renal function in dialysis patients. Kidney Int..

[3] Liu X., Dai C. (2017). Advances in understanding and management of residual renal function in patients with chronic kidney disease. Kidney Dis..

[4] Liao C.T., Chen Y.M., Shiao C.C., Hu F.C., Huang J.W., Kao T.W., Chuang H.F., Hung K.Y., Wu K.D., Tsia T. (2009). Rate of decline of residual renal function is associated with all-cause mortality and technique failure in patients on long-term peritoneal dialysis. Nephrol. Dial. Transpl..

[5] Lang S.M., Bergner A., Töpfer M., Schiffl H. (2001). Preservation of residual renal function in dialysis patients: Effects of dialysis-technique-related factors. Perit. Dial. Int..

[6] Wang J., Xie X., Yan X., Yang X., Zhang X., Chen J., Han F. (2019). A fast decline of residual renal function in the first year is a predictor for early withdrawal from peritoneal dialysis in non-diabetic patients. Kidney Blood Press Res..

[7] Termorshuizen F., Korevaar J.C., Dekker F.W., van Manen J.G., Boeschoten E.W., Krediet R.T., NECOSAD Study Group (2003). The relative importance of residual renal function compared with peritoneal clearance for patient survival and quality of life: An analysis of the Netherlands Cooperative Study on the Adequacy of Dialysis (NECOSAD)-2. Am. J. Kidney Dis..

[8] Marrón B., Remón C., Pérez-Fontán M., Quirós P., Ortíz A. (2008). Benefits of preserving residual renal function in peritoneal dialysis. Kidney Int. Suppl..

[9] Kang S.H., Cho K.H., Park J.W., Yoon K.W., Do J.Y. (2013). Proteinuria as a risk factor for decline in residual renal function in non-diabetic peritoneal dialysis patients. Kidney Blood Press Res..

[10] Cho Y., Johnson D.W., Vesey D.A., Hawley C.M., Clarke M., Topley N. (2015). Utility of urinary biomarkers in predicting loss of residual renal function: The balANZ Trial. Perit. Dial. Int..

[11] Hidaka H., Nakao T. (2003). Preservation of residual renal function and factors affecting its decline in patients on peritoneal dialysis. Nephrology.

[12] Szeto C.C., Kwan B.C., Chow K.M., Chung S., Yu V., Cheng P.M., Leung C.B., Law M.C., Li P.K. (2015). Predictors of residual renal function decline in patients undergoing continuous ambulatory peritoneal dialysis. Perit. Dial. Int..

[13] Federico G., Meister M., Mathow D., Heine G.H., Moldenhauer G., Popovic Z.V., Nordström V., Kopp-Schneider A., Hielscher T., Nelson P.J. (2016). Tubular Dickkopf-3 promotes the development of renal atrophy and fibrosis. JCI Insight.

[14] Zewinger S., Rauen T., Rudnicki M., Federico G., Wagner M., Triem S., Schunk S.J., Petrakis I., Schmit D., Wagenpfeil S. (2018). Dickkopf-3 (DKK3) in urine identifies patients with short-term risk of eGFR loss. J. Am. Soc. Nephrol..

[15] Schunk S.J., Zarbock A., Meersch M., Küllmar M., Kellum J.A., Schmit D., Wagner M., Triem S., Wagenpfeil S., Gröne H.J. (2019). Association between urinary dickkopf-3, acute kidney injury, and subsequent loss of kidney function in patients undergoing cardiac surgery: An observational cohort study. Lancet.

[16] Schunk S.J., Speer T., Petrakis I., Fliser D. (2021). Dickkopf 3-a novel biomarker of the ‘kidney injury continuum’. Nephrol. Dial. Transpl..

[17] Fang X., Hu J., Chen Y., Shen W., Ke B. (2020). Dickkopf-3: Current knowledge in kidney diseases. Front. Physiol..

[18] Uchiyama K., Yanai A., Maeda K., Ono K., Honda K., Tsujimoto R., Kamijo Y., Yanagi M., Ishibashi Y. (2017). Baseline and time-averaged values predicting residual renal function decline rate in Japanese peritoneal dialysis patients. Ther. Apher. Dial..

[19] Seibert F.S., Heringhaus A., Pagonas N., Rohn B., Bauer F., Trappe H.J., Landmesser U., Babel N., Westhoff T.H. (2021). Dickkopf-3 in the prediction of contrast media induced acute kidney injury. J. Nephrol..

[20] MacDonald B.T., Tamai K., He X. (2009). Wnt/beta-catenin signaling: Components, mechanisms, and diseases. Dev. Cell.

[21] Tan R.J., Zhou D., Zhou L., Liu Y. (2014). Wnt/β-catenin signaling and kidney fibrosis. Kidney Int. Suppl..

[22] Sato M., Muragaki Y., Saika S., Roberts A.B., Ooshima A. (2003). Targeted disruption of TGF-beta1/Smad3 signaling protects against renal tubulointerstitial fibrosis induced by unilateral ureteral obstruction. J. Clin. Investig..

[23] Rodríguez-Iturbe B., Johnson R.J., Herrera-Acosta J. (2005). Tubulointerstitial damage and progression of renal failure. Kidney Int. Suppl..

[24] Nath K.A. (1992). Tubulointerstitial changes as a major determinant in the progression of renal damage. Am. J. Kidney Dis..

[25] Johnson D.W., Mudge D.W., Sturtevant J.M., Hawley C.M., Campbell S.B., Isbel N.M., Hollett P. (2003). Predictors of decline of residual renal function in new peritoneal dialysis patients. Perit. Dial. Int..

[26] Yang C., Ma X., Zhao W., Chen Y., Lin H., Luo D., Zhang J., Lou T., Peng Y., Peng H. (2020). A longitudinal analysis of the relationship between serum uric acid and residual renal function loss in peritoneal dialysis patients. Ren. Fail..

[27] Liu X.Y., Gao X.M., Zhang N., Chen R., Wu F., Tao X.C., Li C.J., Zhang P., Yu P. (2017). Oral bicarbonate slows decline of residual renal function in peritoneal dialysis patients. Kidney Blood Press Res..

[28] Tian N., Guo Q., Zhou Q., Cao P., Hong L., Chen M., Yang X., Yu X. (2016). The impact of fluid overload and variation on residual renal function in peritoneal dialysis patient. PLoS ONE.

